# Gastrosplenic Fistula with Thoracic Extension as a Complication of Primary Splenic Lymphoma

**DOI:** 10.5334/jbsr.4186

**Published:** 2026-03-05

**Authors:** Diogo Carvalho, Luis Cabral, João Caldeira

**Affiliations:** 1 Radiology Department, Instituto Português de Oncologia (IPO) de Lisboa Francisco Gentil, Lisbon, Portugal

**Keywords:** diffuse large B-Cell lymphoma, spleen, hydropneumothorax, gastrosplenic fistula, computed tomography (CT)

## Abstract

*Teaching point:* Gastrosplenic fistula, a rare complication of splenic lymphoma, can be diagnosed early with computed tomography (CT), which also delineates diaphragmatic or thoracic extension, facilitating timely management and preventing further thoracoabdominal involvement.

## Case History

**Figure 1:**
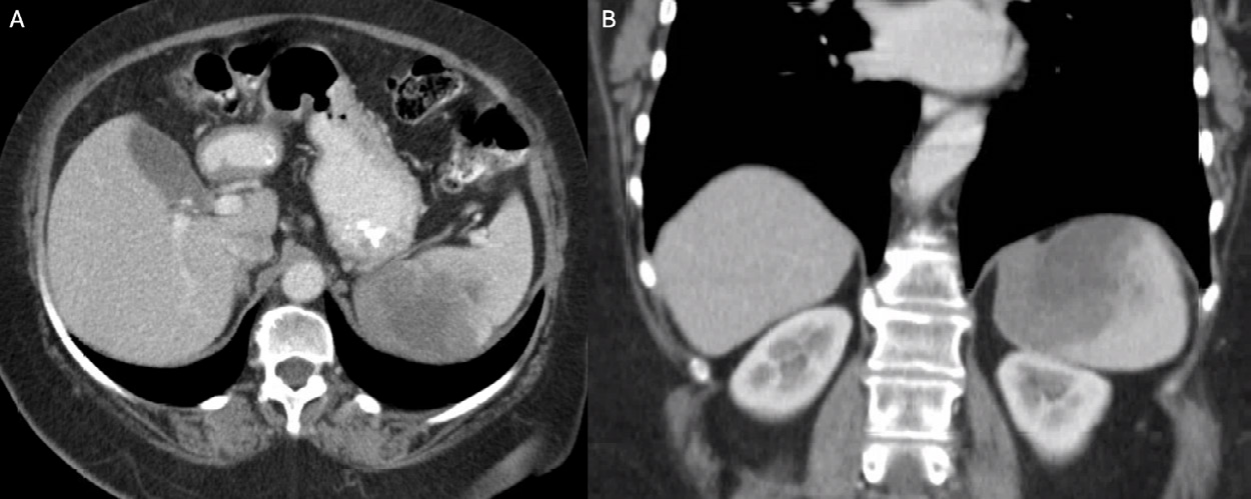
Axial **(A)** and coronal **(B)** contrast‑enhanced CT images obtained two months before the current presentation show an infiltrative hypodense splenic mass abutting the left hemidiaphragm and adjacent to the stomach, without clear signs of invasion.

A 66‑year‑old woman with known primary splenic diffuse large B‑cell lymphoma (Figures 1A and 1B) undergoing chemotherapy presented with acute upper abdominal pain, nausea, vomiting, fever, and sudden dyspnea. Laboratory tests revealed elevated inflammatory markers, including a C‑reactive protein level of 36 mg/dL.

**Figure 2:**
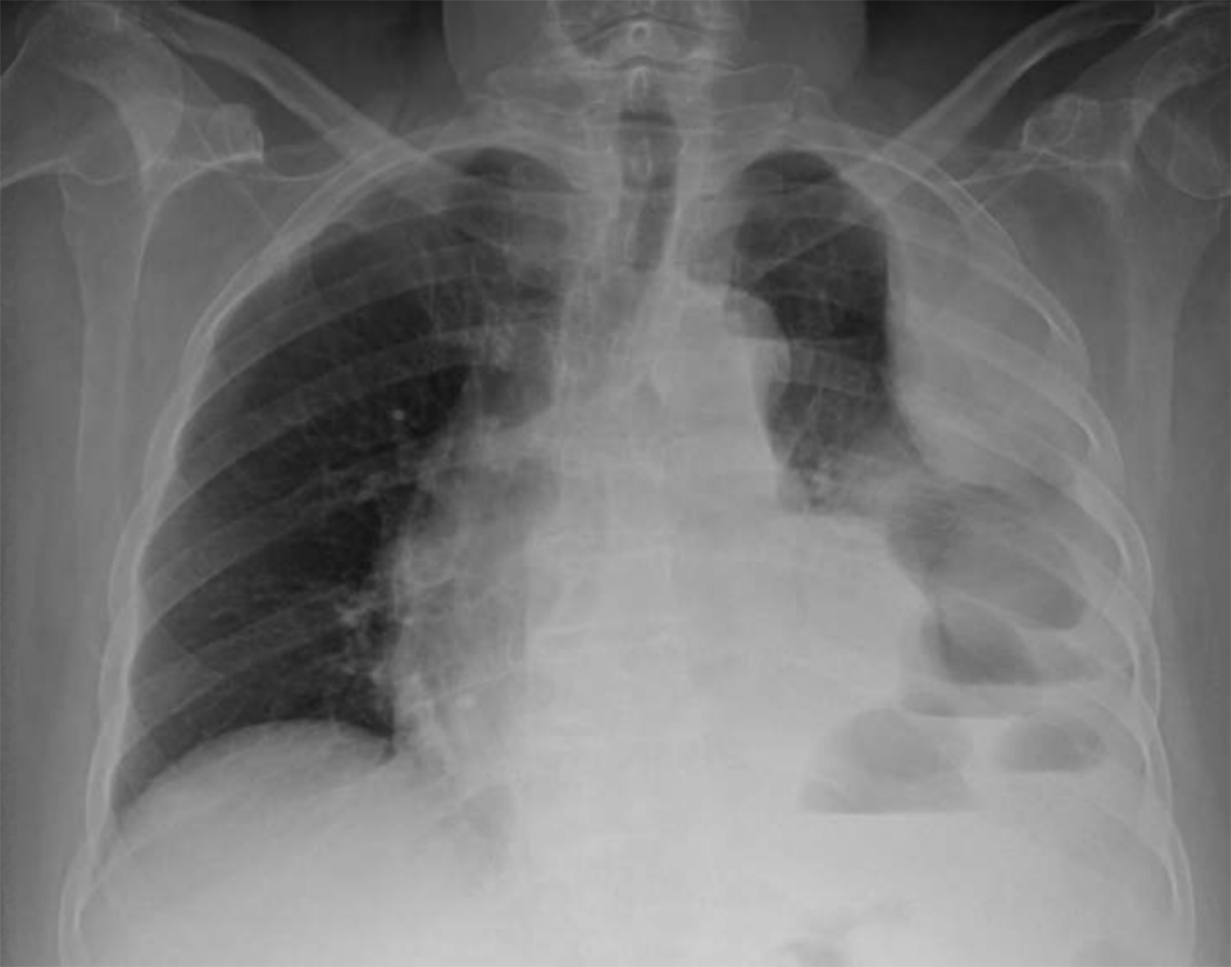
Multiple air–fluid levels in the left hemithorax, consistent with hydropneumothorax.

Chest radiograph (Figure 2) showed a pleural‑based opacity and another opacity abutting the cardio‑mediastinal silhouette in the left hemithorax, accompanied by blunting of the left costophrenic angle and multiple air–fluid levels consistent with hydropneumothorax.

**Figure 3:**
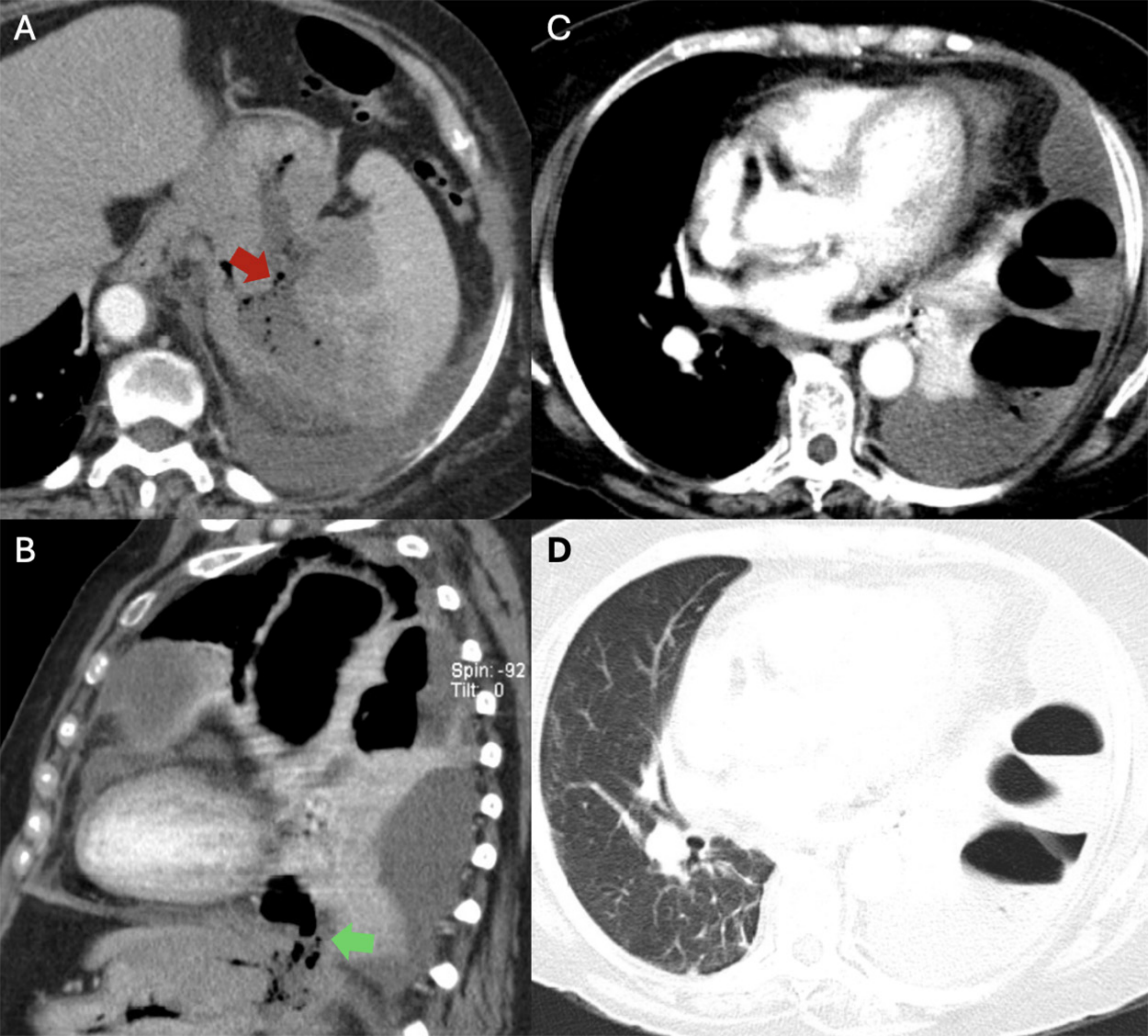
Contrast‑enhanced CT images demonstrate a gastrosplenic fistula (red arrow in **A**) and a diaphragmatic defect (green arrow in **B**), resulting in leakage of gastric contents into the pleural cavity and the development of a multiloculated hydropneumothorax (**C** and **D**).

Contrast‑enhanced computed tomography (CT) demonstrated a fistulous tract between the gastric fundus and the spleen, with leakage of gastric contents into the splenic tissue already extensively infiltrated by lymphoma (Figure 3A). A left diaphragmatic defect (Figure 3B) allowed transdiaphragmatic passage of gastric material into the pleural cavity, producing a large multiloculated hydropneumothorax with air–fluid levels and passive atelectasis of the adjacent lung (Figures 3C and 3D).

These findings confirmed a gastrosplenic fistula caused by a splenic tumor invading the stomach, with subsequent thoracic extension through the left hemidiaphragm.

## Comment

Gastrosplenic fistula (GSF) is a rare, life‑threatening complication most often associated with aggressive lymphomas. It results from direct tumor invasion or rapid necrosis of infiltrated spleen and gastric walls, potentially accelerated by chemotherapy [1].

In this patient, CT provided definitive evidence of a fistulous communication between the gastric fundus and the spleen, complicated by thoracic spread due to a diaphragmatic defect—an exceptionally rare occurrence. Because GSF can mimic a splenic abscess due to intralesional air, CT remains the diagnostic modality of choice, allowing visualization of the fistulous tract and related complications [1].

Treatment usually involves splenectomy with partial or total gastrectomy and diaphragmatic repair when required. Prognosis is poor in the presence of sepsis or advanced disease [1]. This patient developed septic shock and was unfit for surgery. Despite antibiotic therapy and chest drainage, she died a few days later.

This case highlights gastrosplenic fistula as a serious complication of splenic lymphoma and emphasizes that diaphragmatic involvement can allow further extension into the thoracic cavity. Early recognition on imaging is crucial to facilitate timely, potentially lifesaving management.
